# Bifunctional Inhibitors of Influenza Virus Neuraminidase: Molecular Design of a Sulfonamide Linker

**DOI:** 10.3390/ijms222313112

**Published:** 2021-12-03

**Authors:** Sergei Evteev, Dmitry Nilov, Aleksandra Polenova, Vytas Švedas

**Affiliations:** 1 Faculty of Medicine, Lomonosov Moscow State University, Lomonosov Ave. 27, Bldg. 1, 119991 Moscow, Russia; evteevsa1320@gmail.com (S.E.); polenova.alexandra@yandex.ru (A.P.); 2 Belozersky Institute of Physicochemical Biology, Lomonosov Moscow State University, Lenin Hills 1, Bldg. 40, 119991 Moscow, Russia; nilov@belozersky.msu.ru; 3 Research Computing Center, Lomonosov Moscow State University, Lenin Hills 1, Bldg. 4, 119234 Moscow, Russia; 4 Faculty of Bioengineering and Bioinformatics, Lomonosov Moscow State University, Lenin Hills 1, Bldg. 73, 119991 Moscow, Russia

**Keywords:** sulfonamides, 430-cavity, docking, structural filtration

## Abstract

The growing resistance of the influenza virus to widely used competitive neuraminidase inhibitors occupying the active site of the enzyme requires the development of bifunctional compounds that can simultaneously interact with other regulatory sites on the protein surface. When developing such an inhibitor and combining structural fragments that could be located in the sialic acid cavity of the active site and the adjacent 430-cavity, it is necessary to select a suitable linker not only for connecting the fragments, but also to ensure effective interactions with the unique arginine triad Arg118-Arg292-Arg371 of neuraminidase. Using molecular modeling, we have demonstrated the usefulness of the sulfonamide group in the linker design and the potential advantage of this functional group over other isosteric analogues.

## 1. Introduction

**Figure 1:**
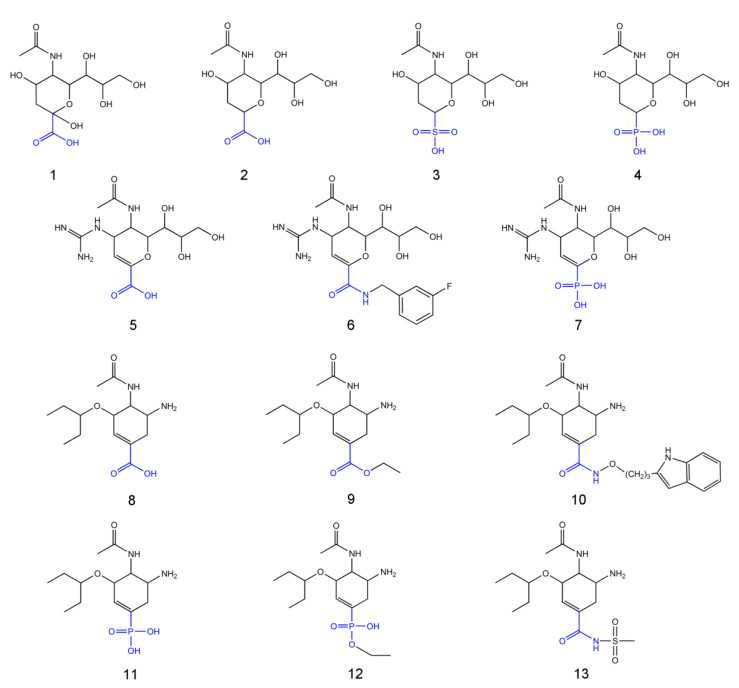
Chemical structures of sialic acid (**1**), deoxysialic acid (**2**), its sulfonic and phosphonic analogues (**3**, **4**), zanamivir (**5**), an amide derivative of zanamivir (**6**), a phosphonic analogue of zanamivir (**7**), oseltamivir carboxylate (**8**), oseltamivir (**9**), a hydroxamic derivative (**10**), a phosphonic analogue of oseltamivir and its ethyl ester (**11**, **12**), and an analogue containing a sulfonamide group (**13**). The functional group that can interact with the arginine triad is highlighted in blue.

Among the known influenza viruses, the most dangerous is type A, which affects approximately 1 billion people annually and causes periodic pandemics [1,2,3]. Influenza A viruses, in turn, are classified according to the antigenic properties of the surface proteins hemagglutinin (H) and neuraminidase (N). Thus, the Spanish flu of 1918, the Asian flu of 1957, the Hong Kong flu of 1968, and the 2009 swine flu pandemic were caused by the subtypes H1N1, H2N2, H3N2 and H1N1pdm09, respectively [4]. Hemagglutinin recognizes terminal sialic acid residues (compound **1** in Figure 1) of oligosaccharide receptors on epithelial cells and provides the penetration of the virus into the cell cytoplasm, while neuraminidase cleaves sialic acid to facilitate the release of newly formed viral particles from the cell surface [5,6].

Neuraminidase is a target for the most effective anti-influenza drugs zanamivir (**5**) and oseltamivir (**9**) [7,8,9]. These molecules compete for the sialic acid binding site formed by a number of charged residues as well as by the side chain of the catalytic residue Tyr406, and thereby suppress the enzyme activity [10,11]. However, inhibitors gradually lose their effectiveness due to a high frequency of mutations in the neuraminidase active site. In the period 2008–2009, there was a dramatic increase in the resistance of the H1N1 virus to oseltamivir due to the H275Y mutation in the sialic acid binding site [12,13]. Other substitutions reducing the inhibitory effect have been also reported: E119A (H5N1) [14], Q136K/R (H1N1), R292K (H3N2) [15,16]. This indicates the need to develop new inhibitors that could bind to other sites on the protein surface and suppress neuraminidase activity.

**Figure 2:**
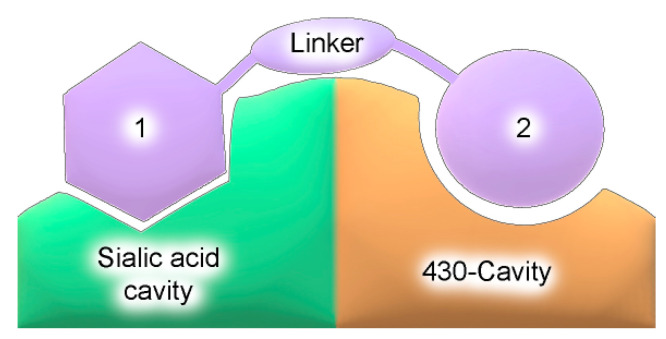
Schematic representation of a hypothetical bifunctional neuraminidase inhibitor. Structural fragments 1 and 2 connected by a linker are bound to different sites on the protein surface.

A possible solution would be to create bifunctional compounds that occupy the sialic acid binding cavity and the adjacent 430-cavity (Figure 2). It is assumed that, when using bifunctional inhibitors, mutations in one of the binding sites may be less critical if effective interactions with another site are maintained, which, in turn, can hinder the selection of resistant mutant strains of the virus [17,18]. This approach allows the creation of more effective inhibitors, the use of which reduces the risk of resistance due to complex interactions with the target protein. The 430-cavity was identified as an additional binding site in N1 and N2 neuraminidases that could be targeted by inhibitors; its hydrophobic surface is formed with conserved residues Pro326, Ile427, and Thr439 [19,20,21,22,23]. The arginine triad Arg118-Arg292-Arg371, located at the interface between the sialic acid binding site and 430-cavity, interacts with the carboxyl group of the sialic acid residue. This interaction plays a key role in the binding of both the substrate and competitive inhibitors of neuraminidase [8,24,25], which should be taken into account when designing bifunctional compounds.

A fundamentally important part of a hypothetical bifunctional neuraminidase inhibitor is a linker which should (i) ensure the optimal position of structural fragments of the molecule in their binding sites and (ii) form hydrogen bonds with the arginine triad, since this interaction makes a significant contribution to the efficiency of binding. It is obvious that the linker design should be based on some functional group, an acceptor of hydrogen bonds. Earlier, derivatives of sialic acid, zanamivir and oseltamivir were obtained, in which the amide, phosphono, or sulfo group occupied a position suitable for interaction with the arginine triad in enzyme-inhibitor complexes [26,27,28,29]. In this work, we investigated the possibility of creating a suitable linker based on the sulfonamide group, the choice of which was due to several factors. Firstly, this functional group is an isosteric analogue of the carboxyl group [30,31] and is potentially capable of forming hydrogen bonds with the arginine triad. Secondly, such a linker will be stable since sulfonamides are resistant to acid, base, and enzymatic hydrolysis [32]. Thirdly, the sulfonamide group has less pronounced acidic properties compared to the carboxyl group, which may facilitate the penetration of the inhibitor through the cell membrane [31].

## 2. Results and Discussion

To better understand how the interaction between the inhibitor’s functional group and arginine triad Arg118-Arg292-Arg371 can occur, sialic acid and known inhibitors [8,26,27,28,33,34,35,36] were docked into the active sites of N1 and N2 neuraminidases: deoxysialic acid (**2**), its sulfonic and phosphonic analogues (**3**, **4**), zanamivir (**5**), an amide derivative of zanamivir (**6**), a phosphonic analogue of zanamivir (**7**), oseltamivir carboxylate (**8**), oseltamivir (**9**), a hydroxamic derivative (**10**), a phosphonic analogue of oseltamivir and its ethyl ester (**11**, **12**), and an analogue containing a sulfonamide group (**13**). The obtained models are shown in Figures S1–S3; the coordinates of sialic acid, zanamivir and oseltamivir carboxylate in the available crystal structures of neuraminidase-inhibitor complexes (4gzq, 1inx, 3b7e, and 3ti6) were used as a control to confirm the correctness of docking.

**Figure 3:**
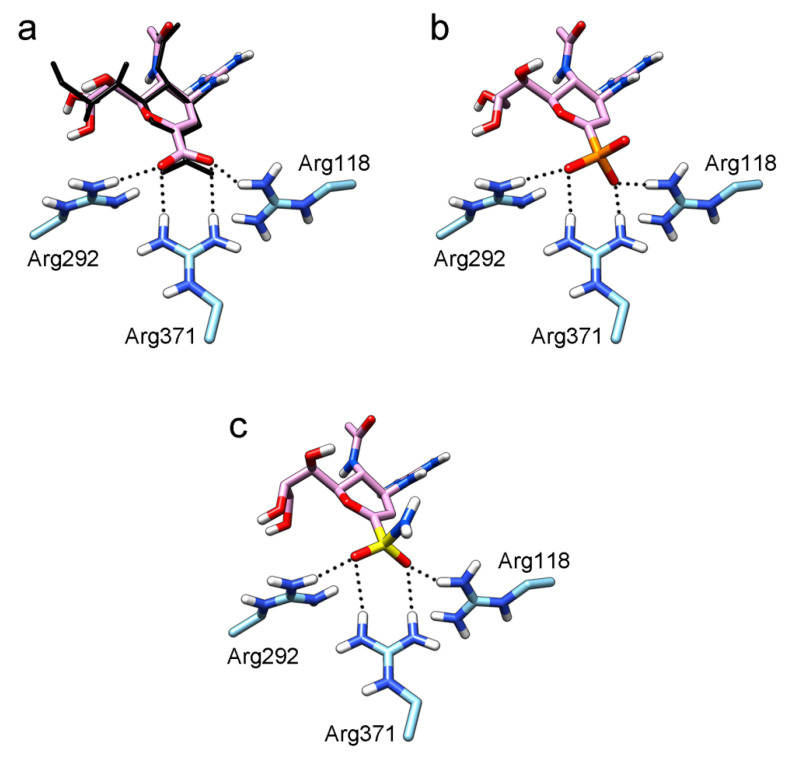
Modeling the interaction of various functional groups of inhibitors with the residues of the arginine triad. (**a**) Zanamivir, (**b**) phosphonic analogue of zanamivir, and (**c**) sulfonamide analogue of zanamivir. The coordinates of zanamivir taken from the available crystal structure 3b7e are shown in black.

In all modeled complexes, the functional group was in contact with the arginine triad, but the efficiency of interaction due to the formed hydrogen bonds was different. The carboxyl group of compounds **1**, **2**, **5**, **8**, and **9** occupied the optimal position for the formation of hydrogen bonds with each of the triad residues (Figure 3a). A similar interaction was observed in the case of the sulfo group of compound **3** and the phosphono group of compounds **4**, **7**, **11**, and **12**: two oxygen atoms acted as acceptors of hydrogen bonds (Figure 3b). In the case of amide derivative **6** and hydroxamic derivative **10**, the functional group lost interaction with one of the triad residues due to the presence of only one acceptor oxygen atom (Supplementary Materials Figures S2b and S3c). The interaction of an analog of oseltamivir containing a sulfonamide fragment (**13**) should be considered separately. In this molecule, the sulfonamide group was connected to cyclohexene scaffold not directly, but through the carbonyl carbon atom, and therefore did not participate in the formation of bonds with the arginine triad (Figure S3f).

Thus, the carboxyl, sulfo, or phosphono groups of the inhibitor can effectively interact with the arginine triad Arg118-Arg292-Arg371 upon binding to the enzyme. However, it should be noted that the corresponding compounds based on these groups will be prone to hydrolysis. As, for example, in the case of oseltamivir (compound **9**), which is a prodrug converted in the body into the active metabolite oseltamivir carboxylate (compound **8**) [37,38]. On the other hand, chemically stable amide and hydroxamic groups cannot provide optimal interaction with the arginine triad, thus indicating the need to continue the search for an optimal linker when creating the suggested bifunctional neuraminidase inhibitor (Table 1).

**Figure 4:**
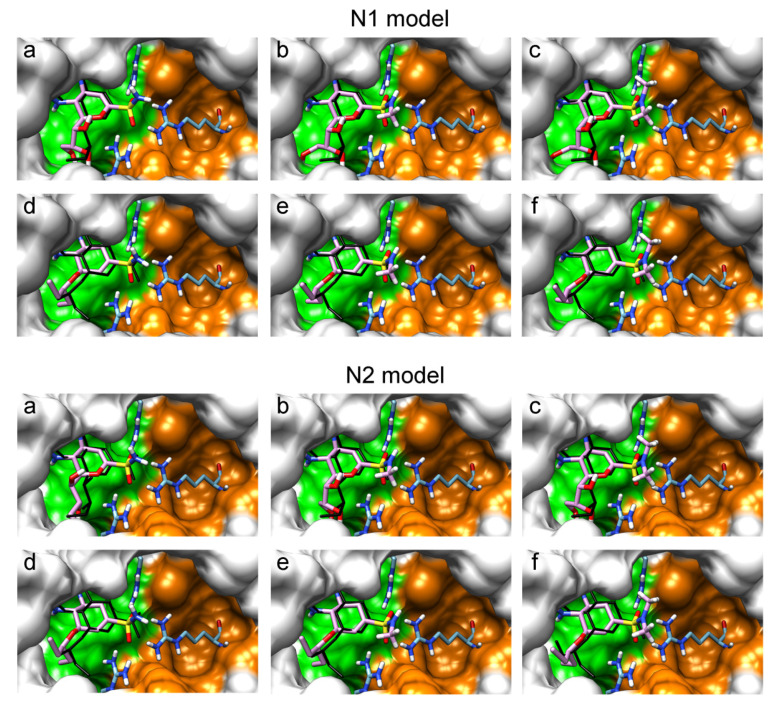
Positions of sulfonamide analogues of zanamivir (**a**–**c**) and oseltamivir (**d**–**f**) in N1 and N2 neuraminidase models. Compounds containing terminal (**a**,**d**), monosubstituted (**b**,**e**) and disubstituted (**c**,**f**) sulfonamide groups are shown. The sialic acid cavity is shown in green, and the 430-cavity in orange. The coordinates of zanamivir and oseltamivir carboxylate taken from the available crystal structures 3b7e and 3ti6 are shown in black.

The sulfonamide group, widely used in medicinal chemistry, can serve as an acceptor of hydrogen bonds and is resistant to hydrolysis [32,39,40]. Hydrogen bonds between the oxygen atom of the sulfonamide fragment of the ligand and the arginine residue can be observed in many protein complexes taken from the Protein Data Bank (Table S1). Interestingly, the Protein Data Bank contains complexes with sulfonamide derivatives of pyranose structurally similar to sialic acid (Table S2), but these compounds were used as inhibitors of carbonic anhydrases and did not interact with arginine residues. To assess the possibility of the formation of key interactions with the arginine triad Arg118-Arg292-Arg371, the structures of sulfonamide analogues of known neuraminidase inhibitors were modeled and docked into the active site of N1 and N2. Figure 4 shows poses of zanamivir and oseltamivir analogues with terminal (unsubstituted), methylated, and dimethylated sulfonamide groups. All compounds are optimally positioned in the sialic acid binding site and form four hydrogen bonds with the arginine triad (Figure 3c, Table S3). Poses in the active site of N1 and N2 neuraminidases are very similar, what can be explained by the strong similarity of the binding sites. The introduction of methyl substituents has no significant effect on the position of the functional group as well as on calculated binding energy (Table S4), suggesting the possibility of further elongation of the inhibitor’s structure towards the 430-cavity.

**Figure 5:**
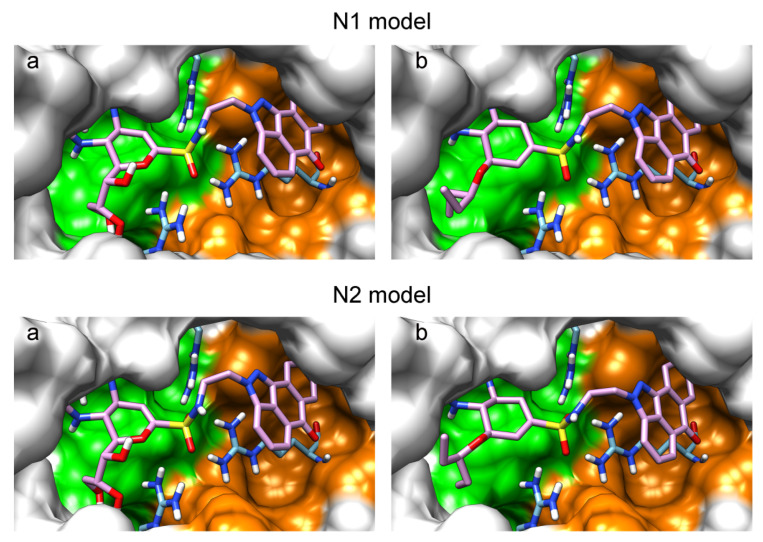
Positions of prototype bifunctional inhibitors based on zanamivir (**a**) and oseltamivir (**b**) in N1 and N2 neuraminidase models. The sulfonamide linker provides the effective interaction with the arginine triad.

An anthrapyrazole derivative was identified in our lab as a complementary ligand for the 430-cavity (experimental results will be published elsewhere). This allows the design of prototype bifunctional inhibitors of neuraminidase by combining the structures of zanamivir/oseltamivir and the anthrapyrazole moiety with a suitable linker. Figure 5 shows the positions of corresponding compounds containing the sulfonamide linker –SO_2_NH–(CH_2_)_2_– in the active site of N1 and N2 neuraminidases. As expected, the anthrapyrazole fragment of the inhibitor is located in the hydrophobic 430-cavity, and the sulfonamide group forms the required interaction with the arginine triad. The introduction of an additional structural fragment significantly raises the calculated binding energy, thus confirming that the chosen approach to inhibitor design is promising (Figure S4, Table S5). The reported resistance mutations (H275Y, E119A, Q136K/R, R292K) are located in the sialic acid cavity and thus may be less critical for the binding of bifunctional sulfonamide inhibitors, the elongated structure of which can interact not only with the sialic acid binding site, but also with the 430-cavity.

Molecular modeling has demonstrated that sulfonamide derivatives of zanamivir and oseltamivir can effectively bind to the active site of neuraminidase. However, other structural fragments complementary to the sialic acid binding site and 430-cavity may be also of interest for the design of new neuraminidase inhibitors. We performed virtual screening of a large set of low-molecular-weight sulfonamides from the ZINC12 library (331516 compounds; 250 ≤ *M_r_* ≤ 350, log *P* ≤ 3.5, rotatable bonds ≤ 7) against the N1 model. As result, 492 compounds were selected that formed effective interactions with the arginine triad: single hydrogen bonds with Arg118 and Arg292, and two bonds with Arg371.

**Figure 6:**
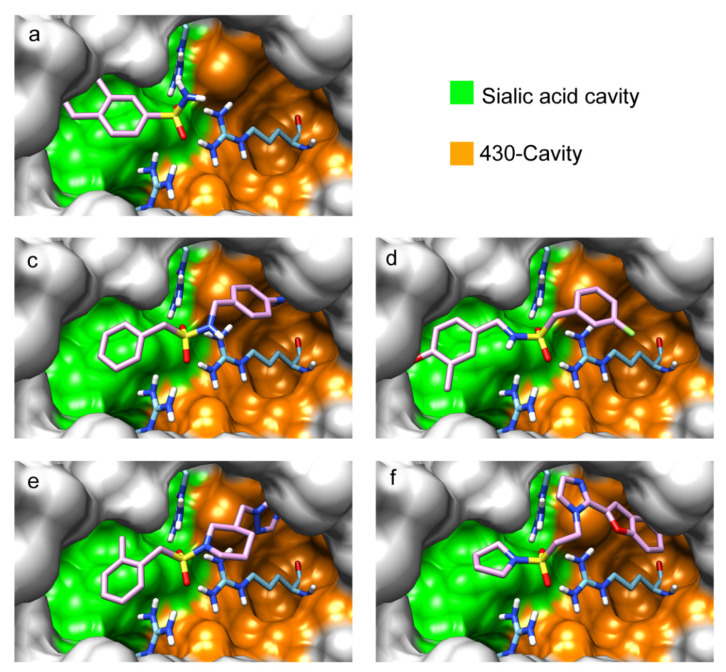
Typical positions of unsubstituted (**a**), monosubstituted (**c**,**d**) and disubstituted (**e**,**f**) sulfonamides selected by virtual screening of the ZINC12 library against N1 neuraminidase. **a**—ZINC13686935, **c**—ZINC13673669, **d**—ZINC68909486, **e**—ZINC97048161, **f**—ZINC77458684. Designations **a**–**f** correspond to orientations of molecules presented in Table 2.

The selected unsubstituted, monosubstituted and disubstituted sulfonamides can be divided into groups according to the orientation of the S–N bond in the N1 active site. In the first case, the S–N bond is oriented towards the 430-cavity (as in the analogues of zanamivir and oseltamivir); in the second, towards the sialic acid binding site (Table 2, Figure 6). Monosubstituted and disubstituted compounds can be oriented in both directions thus providing more opportunities for the design of sulfonamide inhibitors. Interestingly, the incorporation of the nitrogen atom into the heterocycle does not prevent binding both in the sialic acid cavity and 430-cavity (Figure 6e,f). Docking poses of the selected sulfonamides in the active site of the N2 model were very similar to N1 (Figure S5).

Due to rotation around the S–N bond, the sulfonamide group can exist in different conformations, which are defined by the dihedral angle C1–S–N–C2 for monosubstituted compounds, and by angles C1–S–N–C2 and C1–S–N–C3 for disubstituted compounds. In the case of an effective bifunctional neuraminidase inhibitor, the structural fragments should be located in the corresponding cavities of the enzyme, and the linker sulfonamide group should be in an energetically acceptable configuration. Analysis of the docked ZINC12 compounds showed that the position of the sulfonamide group in the N1 active site corresponds to the most common sulfonamide conformations in protein structures from the Protein Data Bank (Figures S6 and S7). In the case of monosubstituted ZINC12 compounds, the C1–S–N–C2 angle was 60–100°; in the case of disubstituted compounds, the C1–S–N–C2 (smaller) angle was 80–90°, and C1–S–N–C3 (larger) angle 90–100°. At these angles, the sulfonamide group is in an energetically favorable staggered conformation [41], and thus its position in the neuraminidase active site can be considered optimal.

## 3. Materials and Methods

Molecular models of neuraminidase were constructed on the basis of crystal structures using Amber 12 (https://ambermd.org, accessed on 1 November 2021) [42]. N1 model was constructed based on the 3b7e structure (complex with zanamivir) [43]. Hydrogen atoms were added considering ionization of amino acid residues, and the protein was solvated by a layer of TIP3P water. Covalent bonds between cysteine residues were specified manually (disulfide bridges 92–417, 124–129, 183–230, 232–237, 278–291, 280–289, 318–336 и 421–447. 92–417, 124–129, 183–230, 232–237, 278–291, 280–289, 318–336, and 421–447). The energy minimization of the obtained system (2500 steepest descent steps + 2500 conjugate gradient steps) was performed using positional restraints on heavy atoms of protein and inhibitor. The Amber-compatible force fields *ff99SB* and *GAFF* [44,45] were used to describe the protein and inhibitor with molecular mechanics. Water and zanamivir molecules were removed from the optimized system to obtain the protein model for docking. N2 neuraminidase model was built based on the 3tic structure [46] using the same methodology.

**Figure 7:**
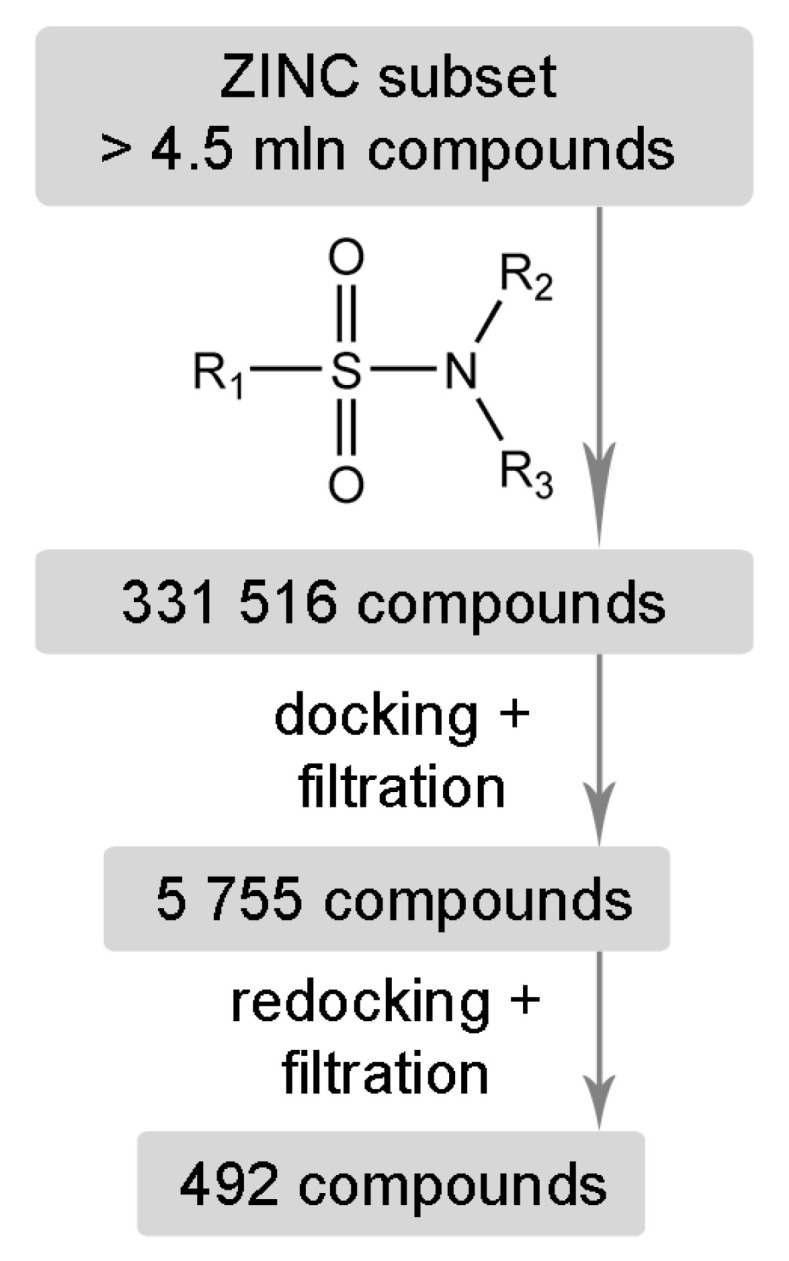
Schematic representation of virtual screening of compounds from the ZINC12 library against N1 neuraminidase.

3D structures of known inhibitors and their derivatives were generated using the CORINA Classic Web service [47]. Compounds were docked into the active site of the N1 and N2 models with Lead Finder 1.1.16, in ‘extra precision’ mode [48,49]. The center of an energy grid box corresponded to carbon coordinates in the carboxyl group of zanamivir, and lengths of box edges were 25 Å. Virtual screening was performed among low-molecular-weight compounds of the ZINC12 library (‘clean leads’ subset) [50,51], as shown in Figure 7. Molecules containing sulfonamide groups were retrieved using a substructure search in ACD/Spectrus DB 14.0 (https://www.acdlabs.com, accessed on 1 November 2021). Sulfonamides were docked into the active site of N1 model in standard mode of Lead Finder, and the obtained poses were subjected to structural filtration with vsFilt [52] to select compounds capable of forming hydrogen bonds with each residue of the arginine triad (Arg118, Arg292, Arg371). The following structural criteria were applied: the distance between guanidinium nitrogen and sulfonamide oxygen ≤ 3.1 Å, the corresponding angle N–H⋯O ≥ 150°. The selected compounds were redocked in the more rigorous mode ‘extra precision’ and were subjected to a more thorough structural filtration. An additional requirement at this stage was the presence of two hydrogen bonds with the Arg371 guanidinium group.

Conformational analysis of the sulfonamide group of low-molecular-weight ligands in the Protein Data Bank and of docked ZINC12 compounds was performed using an in-house Pearl script. The dihedral angle C1–S–N–C2 was measured for monosubstituted sulfonamides, and the angles C1–S–N–C2 and C1–S–N–C3 were measured for disubstituted sulfonamides. UCSF Chimera 1.11.2 (https://www.cgl.ucsf.edu/chimera, accessed on 1 November 2021) was used for the visualization of molecules [53].

## 4. Conclusions

This study has demonstrated that the sulfonamide group is well suited for the construction of a linker in bifunctional neuraminidase inhibitors. It offers potential advantages over other groups (carboxyl, amide, hydroxamic, sulfo, and phosphono), since, along with resistance to hydrolysis, it can be optimally positioned in the active site of N1 and N2 neuraminidases. Docking and virtual screening showed that the sulfonamide linker forms a network of hydrogen bonds with the arginine triad Arg118-Arg292-Arg371 and can be used to combine various structural fragments located in the sialic acid cavity and adjacent 430-cavity into a single structure of a new type of neuraminidase inhibitor.

## Figures and Tables

**Table 1:** Functional groups that can be used to construct a linker in a bifunctional neuraminidase inhibitor.

Functional Group	Linker Structure	Optimal Interaction ^1^	Resistance to Hydrolysis
Carboxyl	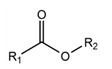	+	−
Amide	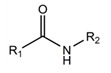	−	+
Hydroxamic	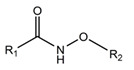	−	+−
Sulfo	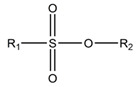	+	−
Phosphono	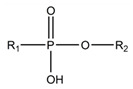	+	−
Sulfonamide	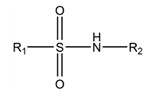	???	+

^1^ Interaction with the arginine triad Arg118-Arg292-Arg371.

**Table 2:** Possible orientations of sulfonamides in the active site of neuraminidase: (**a**) and (**b**) unsubstituted, (**c**) and (**d**) monosubstituted, (**e**) and (**f**) disubstituted compounds. Green color represents the sialic acid cavity, orange represents the 430-cavity. For each variant, the number of sulfonamides selected by virtual screening of the ZINC12 library against N1 model is indicated (number in parentheses refers to disubstituted sulfonamides in which the nitrogen atom is incorporated into the heterocycle).

**a**	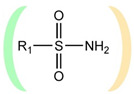	31	**b**	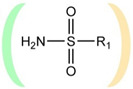	0
**c**	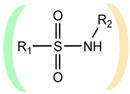	214	**d**	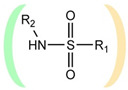	59
**e**	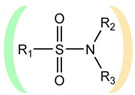	122 (46)	**f**	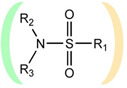	66 (63)

## Data Availability

Not applicable.

## Supplementary Materials

The following are available online at https://www.mdpi.com/article/10.3390/ijms222313112/s1.
