# Supplementary material for: Bifunctional Inhibitors of Influenza Virus Neuraminidase: Molecular Design of a Sulfonamide Linker
Source: Int J Mol Sci. 2021 Dec 3;22(23):13112. doi: 10.3390/ijms222313112 (PMC8657994; doi:10.3390/ijms222313112)
Supplement: Supplementary file 1 [file ijms-22-13112-s001.zip › ijms-1466929-supplementary materials .pdf]

SUPPLEMENT

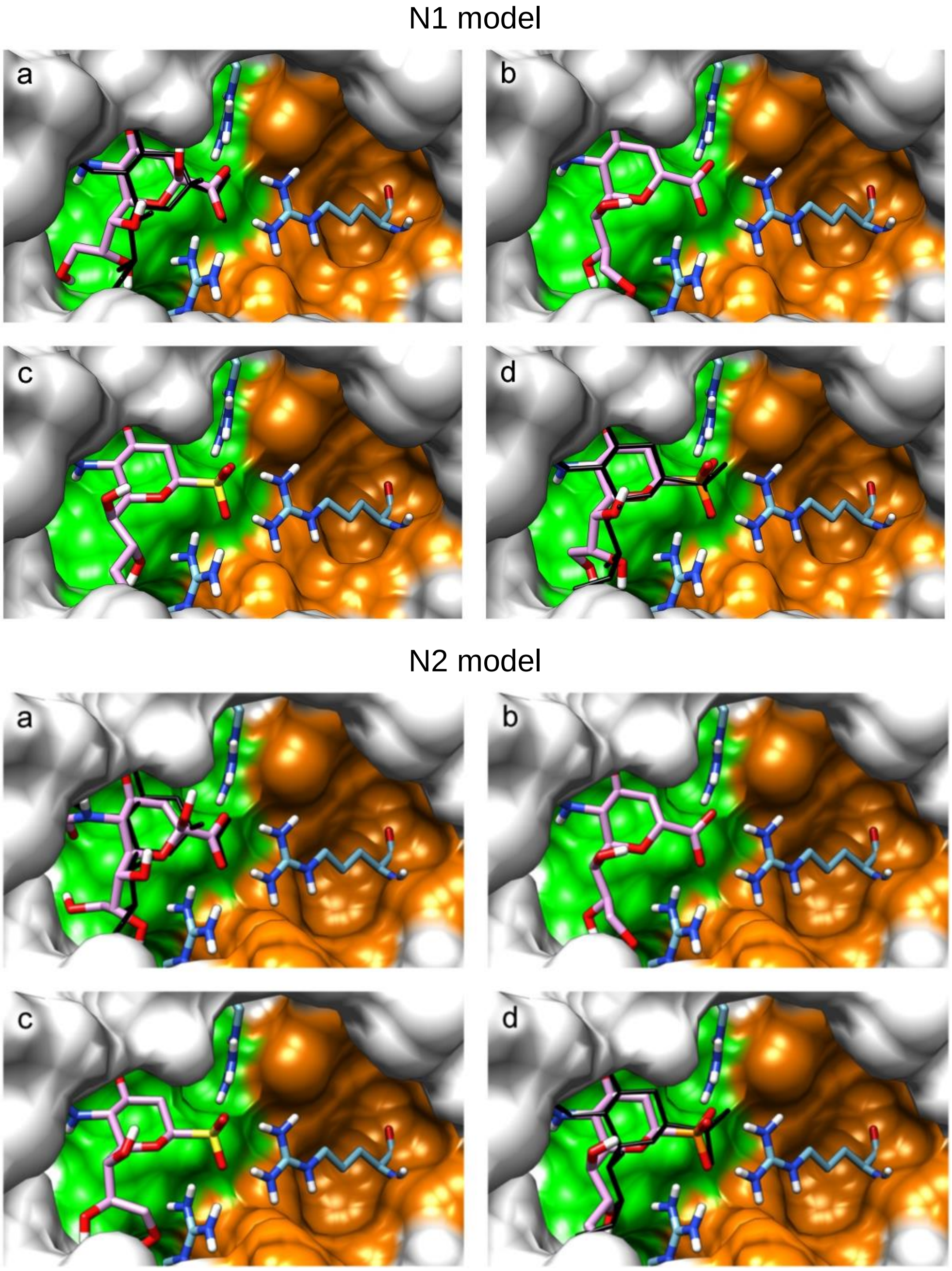

Figure S1. Positions of sialic acid and its analogues in N1 and N2 neuraminidase models. (**a**) Sialic acid **1**, (**b**) deoxysialic acid **2**, (**c**) sulfonic analogue **3**, (**d**) phosphonic analogue **4**. The sialic acid cavity is shown in green, and the 430-cavity in orange. The coordinates of sialic acid and phosphonic analogue taken from the available crystal structures 4gzq and 1inx are shown in black.

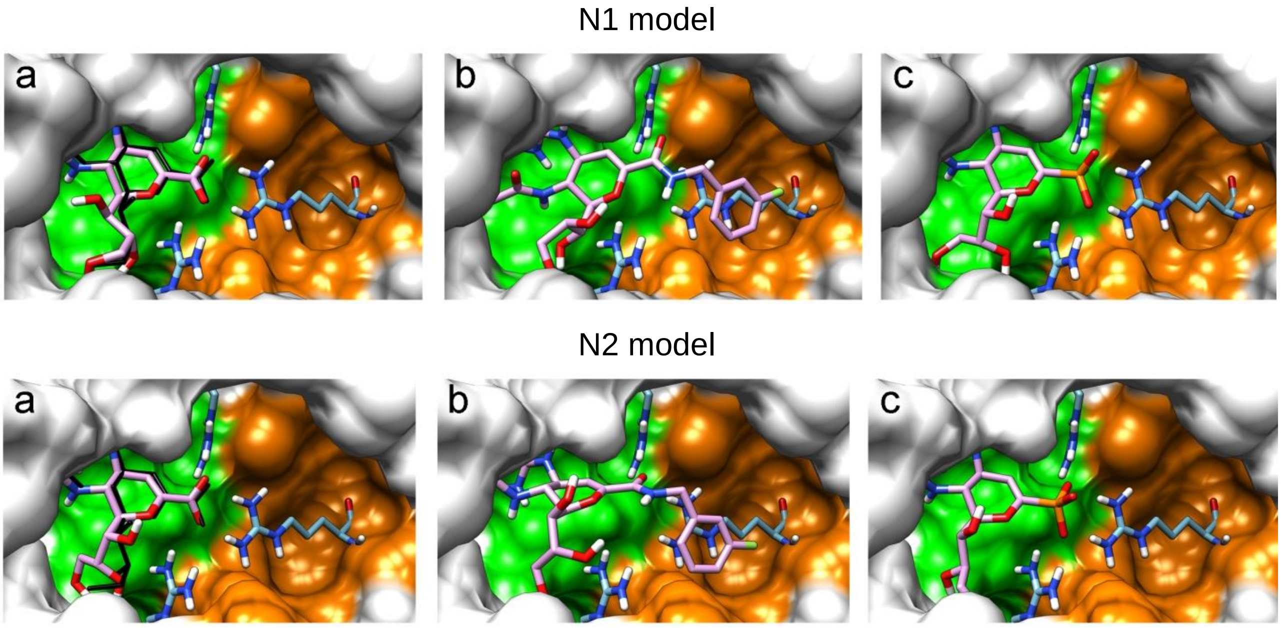

Figure S2. Positions of zanamivir and its analogues in N1 and N2 neuraminidase models. (**a**) Zanamivir **5**, (**b**) amide derivative **6**, (**c**) phosphonic analogue **7**. The coordinates of zanamivir taken from the available crystal structure 3b7e are shown in black.

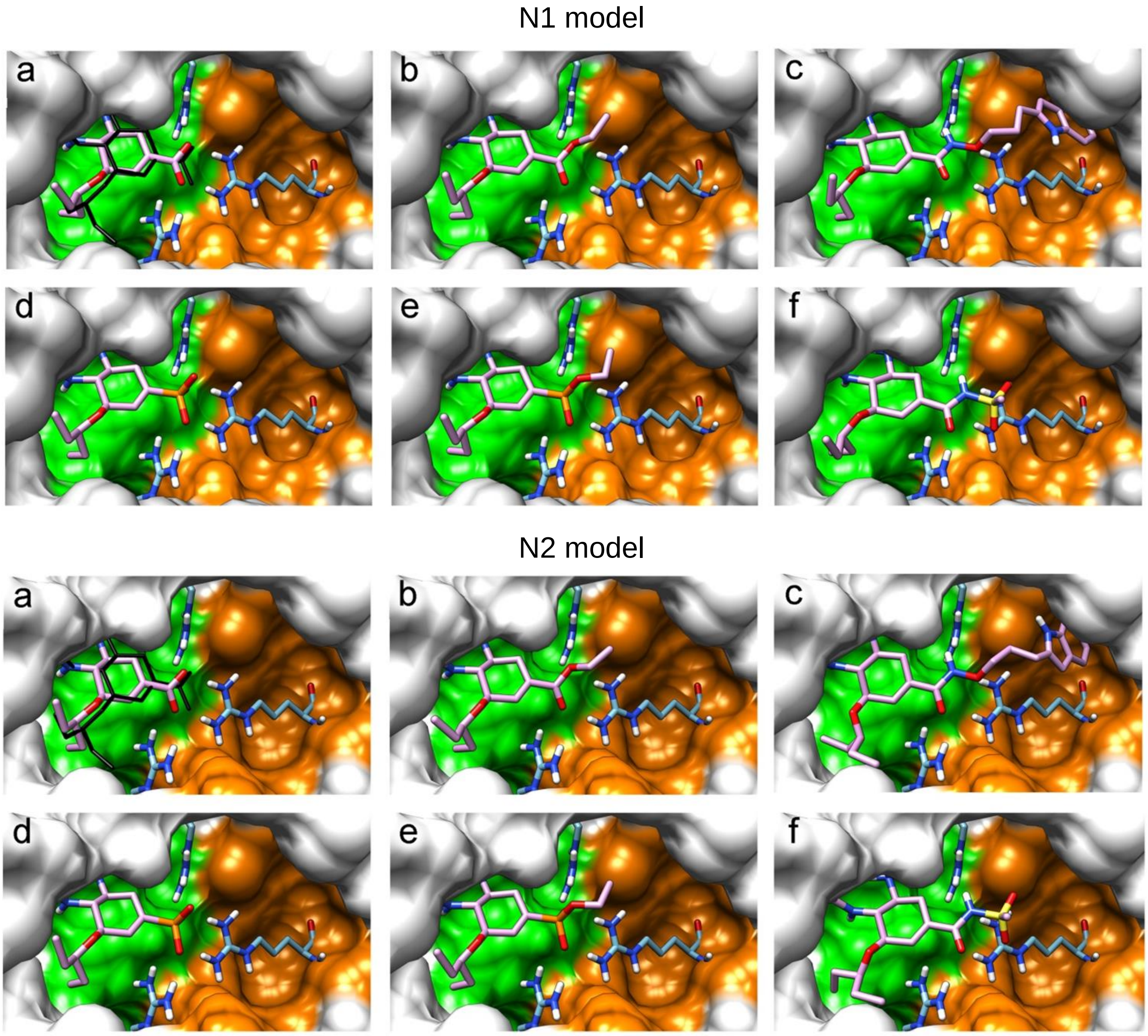

Figure S3. Positions of oseltamivir and its analogues in N1 and N2 neuraminidase models. (**a**) Oseltamivir carboxylate **8**, (**b**) oseltamivir **9**, (**c**) hydroxamic derivative **10**, (**d**) phosphonic analogue **11**, (**e**) ethyl ester of phosphonic analogue **12**, (**f**) analogue with sulfonamide group **13**. The coordinates of oseltamivir carboxylate taken from the available crystal structure 3ti6 are shown in black.

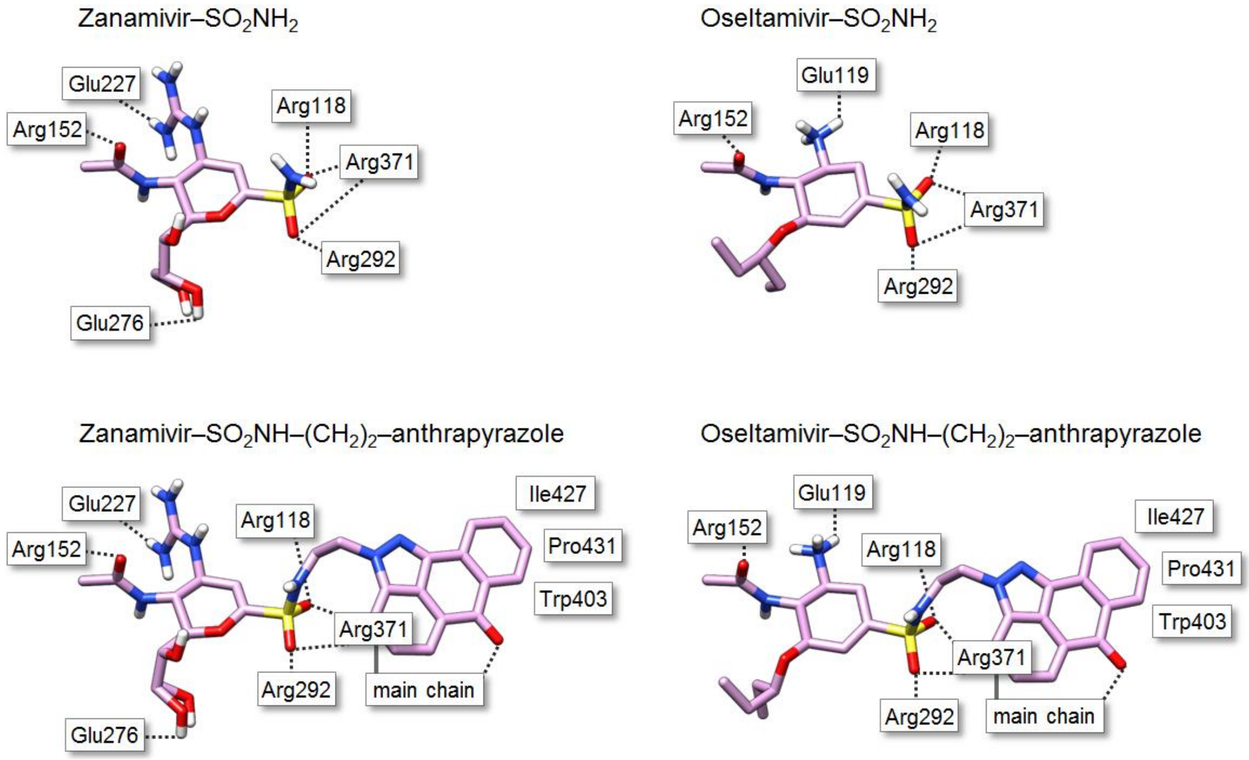

Figure S4. Interaction profiles of prototype bifunctional inhibitors of neuraminidase. The introduction of an anthrapyrazole moiety results in additional interactions with the 430-cavity residues.

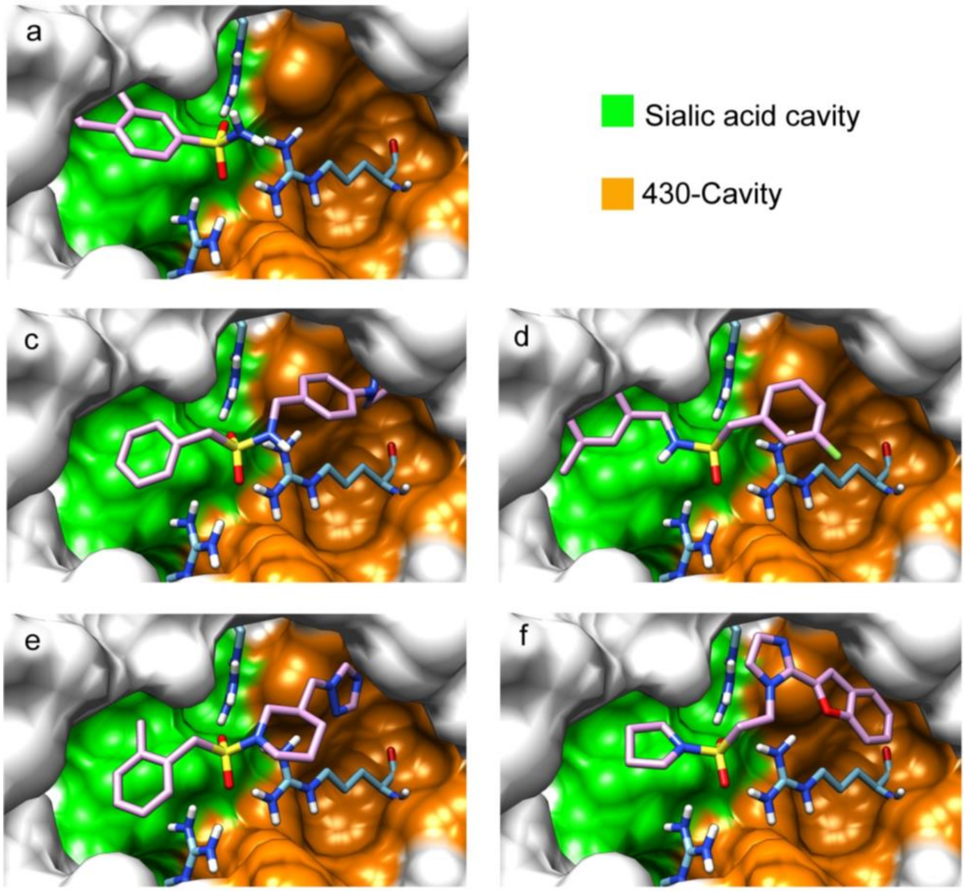

Figure S5. Positions of unsubstituted (**a**), monosubstituted (**c**, **d**) and disubstituted (**e**, **f**) sulfonamides from the ZINC12 library in N2 neuraminidase model. **a** - ZINC13686935, **c** - ZINC13673669, **d** - ZINC68909486, **e** - ZINC97048161, **f** - ZINC77458684.

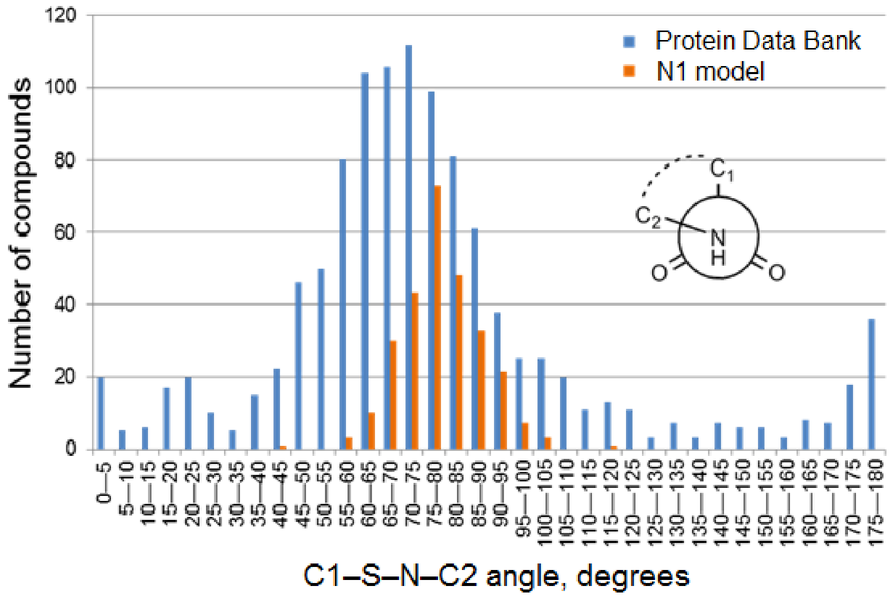

Figure S6. Conformational analysis of monosubstituted sulfonamides in Protein Data Bank structures and in modeled neuraminidase N1 complexes.

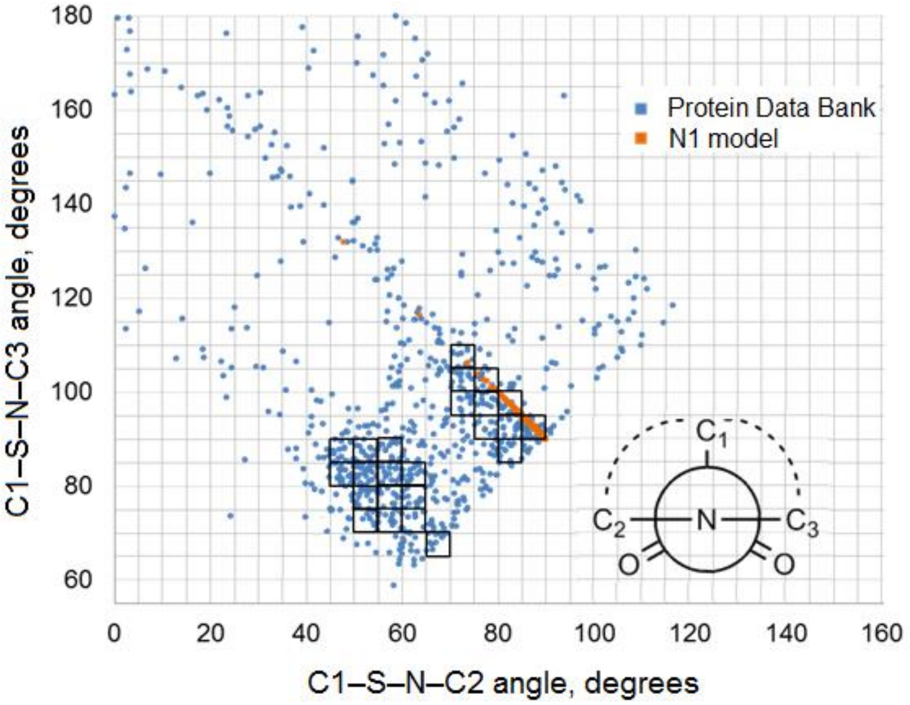

Figure S7. Conformational analysis of disubstituted sulfonamides in Protein Data Bank structures and in modeled neuraminidase N1 complexes. The smaller angle is designated as C1–S–N–C2, and the larger angle as C1–S–N–C3. Each point corresponds to an individual structure; the most occupied areas are marked black.

Table S1. Structures of protein complexes in the Protein Data Bank, in which the terminal (unsubstituted), monosubstituted, or disubstituted sulfonamide group of ligands forms a hydrogen bond with the guanidine group of the arginine residue.

Ligand's formula	PDB ID
RSO_2NH_2	3rd9, 5dpv, 5dt0, 5obr
RSO_2NHR'	1nmq, 1w12, 2d3z, 2d41, 2hwh, 2o5k, 2oz5, 2piy, 2pj7, 2pj8, 2pjb, 2veu, 2xvu, 2xyj, 3lu8, 3n8x, 3qch, 3qci, 3qcj, 3sru, 3wiy, 3zk6, 4bv5, 4bvc, 4fk6, 4hwt, 4ju3, 4ju4, 4muh, 4tlr, 4tpt, 4ury, 5a5i, 5ek0, 5fc4, 5fdo, 5h08, 5ja2, 5khx, 5ml3, 5ml4, 5ml8, 5nal, 5nxd, 5qan, 5t3d, 5vs5, 6baa
$RSO_2NR'R''$	3fqk, 3s53, 4ieh, 5hn7, 5vdi

Table S2. Structures in the Protein Data Bank containing sulfonamide derivatives of pyranose.

Ligand's formula	PDB ID
RSO_2NH_2	3hkn, 3hkq, 3hkt
RSO_2NHR'	4zwx, 4zwy
$RSO_2NR'R''$	4r59, 4r5a, 4r5b, 4zwi, 4zwz, 4zx0, 4zx1

Table S3. Interactions of the sulfonamide group of zanamivir and oseltamivir analogues with residues of the arginine triad in N1 and N2 neuraminidase models. Mean distances (Å), obtained via 10 docking runs, are presented. R′ = R′′ = CH_3. It is assumed that a hydrogen bond between the sulfonamide group and amino group is formed at a distance of ≤ 3.6 Å (Gushchina et al. *J. Chem. Inf. Model.* **2020**, *60*, 3692–3696).

**N1 model**						
Distance	Zanamivir analogues			Oseltamivir analogues		
	RSO_2NH_2	RSO_2NHR'	$RSO_2NR'R''$	RSO_2NH_2	RSO_2NHR'	$RSO_2NR'R''$
SO_2N:O ··· Arg118:NH1	2.80	2.69	2.70	2.80	2.68	2.72
SO_2N:O ··· Arg292:NH2	2.82	2.79	2.76	2.82	2.82	2.76
SO_2N:O ··· Arg371:NH1	3.24	3.37	3.33	3.06	3.20	3.14
SO_2N:O ··· Arg371:NH2	3.25	3.06	3.08	3.24	3.03	2.99
**N2 model**						
Distance	Zanamivir analogues			Oseltamivir analogues		
	RSO_2NH_2	RSO_2NHR'	$RSO_2NR'R''$	RSO_2NH_2	RSO_2NHR'	$RSO_2NR'R''$
SO_2N:O ··· Arg118:NH1	2.79	2.76	2.74	2.83	2.81	2.80
SO_2N:O ··· Arg292:NH2	2.92	2.91	2.81	2.85	2.85	2.83
SO_2N:O ··· Arg371:NH1	3.19	3.25	3.17	3.02	3.02	3.01
SO_2N:O ··· Arg371:NH2	3.16	3.07	2.99	3.11	2.99	3.03

Table S4. Calculated binding energy of sulfonamide analogues of zanamivir and oseltamivir docked into the active site of N1 and N2 neuraminidase models. R′ = R′′ = CH_3. Mean values of ΔG, obtained via 10 docking runs, are presented with standard deviations. K_i values were calculated using formula $\Delta G = RT \ln(K_i)$.

Tested compound		ΔG^{calc}, kcal/mol		K_i^{calc}, nM	
		**N1 model**	**N2 model**	**N1 model**	**N2 model**
Zanamivir analogues	RSO_2NH_2	−11.83 ± 0.27	−11.78 ± 0.14	2.4	2.6
	RSO_2NHR'	−11.76 ± 0.17	−11.66 ± 0.15	2.7	3.2
	$RSO_2NR'R''$	−11.55 ± 0.21	−11.55 ± 0.08	3.9	3.9
Oseltamivir analogues	RSO_2NH_2	−9.28 ± 0.07	−9.15 ± 0.16	174	216
	RSO_2NHR'	−9.13 ± 0.07	−9.09 ± 0.11	223	239
	$RSO_2NR'R''$	−9.23 ± 0.03	−9.19 ± 0.21	189	202

Table S5. Calculated binding energy of prototype bifunctional inhibitors docked into the active site of N1 and N2 neuraminidase models. Mean values of ΔG, obtained via 10 docking runs, are presented with standard deviations. K_i values were calculated using formula $\Delta G = RT \ln(K_i)$.

Tested compound	ΔG^{calc}, kcal/mol		K_i^{calc}, nM	
	**N1 model**	**N2 model**	**N1 model**	**N2 model**
Zanamivir–SO_2NH_2	−11.83 ± 0.27	−11.78 ± 0.14	2.4	2.6
Zanamivir–SO_2NH–$(CH_2)_2$–anthrapyrazole	−14.32 ± 0.22	−14.49 ± 0.51	0.04	0.03
Oseltamivir–SO_2NH_2	−9.28 ± 0.07	−9.15 ± 0.16	174	216
Oseltamivir–SO_2NH–$(CH_2)_2$–anthrapyrazole	−12.08 ± 0.48	−11.46 ± 0.36	1.6	4.5
